# Improved Detection Sensitivity of Spring Viremia of Carp Virus by Substituting a Two-Step with a One-Step Nested Reverse Transcription Polymerase Chain Reaction Method

**DOI:** 10.3390/microorganisms13122727

**Published:** 2025-11-29

**Authors:** Ji-Yoon Park, In-Joo Shin, Hyunwoo Kim, Eun Sup Lee, Euna Choi, Hyoung Jun Kim, Se Ryun Kwon

**Affiliations:** 1 Department of Aquatic Life Medical Sciences, Sunmoon University, Asan 31460, Republic of Korea; yoon3740@naver.com (J.-Y.P.); carol1790@naver.com (I.-J.S.); rlagusdn4327@naver.com (H.K.); dldmstjq1959@naver.com (E.S.L.); 2 WOAH Reference Laboratory for VHS, National Institute of Fisheries Science, Pusan 46083, Republic of Korea; choiea98@pusan.ac.kr (E.C.); hjkim1882@korea.kr (H.J.K.)

**Keywords:** spring viremia of carp virus, one-step semi-nested RT-PCR, detection sensitivity, diagnostic method

## Abstract

Spring viremia of carp (SVC) is a highly contagious disease that affects cyprinids, resulting in systemic hemorrhage, abdominal distension, exophthalmia, and high mortality in juveniles. This can lead to significant losses in the aquaculture industry. The World Organization for Animal Health (WOAH) recommends a two-step semi-nested reverse transcription polymerase chain reaction (RT-PCR) method for diagnosis. However, this method is labor-intensive, requires large reagent volumes, and is prone to carry-over contamination. Here, we evaluated the detection sensitivity of one-step semi-nested RT-PCR (combining RT and primary amplification in a single tube, followed by a second nested PCR step) against conventional two-step semi-nested RT-PCR. SVC virus (SVCV) subgroup Ia was tested using cell culture, RT-quantitative PCR, and one-step RT-PCR. The two-step semi-nested PCR method detected viral RNA up to a 10^−2^ dilution, whereas one-step semi-nested RT-PCR detected it up to a 10^−5^ dilution, showing a 1000-fold improvement in sensitivity. Moreover, detection rates increased from 84.2% with two-step semi-nested RT-PCR to 91.7% with one-step semi-nested RT-PCR in fish tissue samples. One-step semi-nested RT-PCR reduces processing time, minimizes handling steps, and contamination risk, and enhances analytical sensitivity. This supports its adoption as a practical, high-throughput diagnostic tool for SVCV and consideration for future WOAH guidelines.

## 1. Introduction

Spring viremia of carp (SVC) is a viral disease recognized by the World Organization for Animal Health (WOAH) and subject to statutory control in the Republic of Korea. SVC outbreaks predominantly occur during the spring at water temperatures between 10 and 17 °C. This disease is caused by the SVC virus (SVCV), a negative-sense, single-stranded RNA virus that belongs to the genus *Sprivivirus* within the family *Rhabdoviridae*. The genome of SVCV is approximately 11 kb in length and organized into five genes in the order 3′-N-P-M-G-L-5′ [1].

SVCV mainly infects cyprinids belonging to the family *Cyprinidae*, including the common carp (*Cyprinus carpio*), grass carp (*Ctenopharyngodon idella*), silver carp (*Hypophthalmichthys molitrix*), and goldfish (*Carassius auratus*). It can also infect some non-cyprinids, including the European catfish (*Silurus glanis*), leading to substantial economic losses [2].

Infected carp show clinical signs, including petechial hemorrhage, abdominal distention, and exophthalmia, with juvenile mortality reaching 90% [3,4]. The WOAH outlined methods in its Aquatic Manual for SVCV detection that include conventional reverse transcription polymerase chain reaction (RT-PCR), real-time RT-PCR, in situ hybridization, immunohistochemistry, enzyme-linked immunosorbent assay (ELISA), and indirect fluorescent antibody testing (IFAT) [3]. Real-time RT-PCR is highly sensitive; however, it requires specialized equipment, trained personnel, and relatively expensive reagents, including SYBR Green or hydrolysis probes [5]. Serological assays, including ELISA and IFAT, can be labor-intensive and time-consuming, and they may exhibit cross-reactivity [6].

According to the WOAH manual, the semi-nested RT-PCR method developed by Stone et al. [7] remains the most widely used and recommended diagnostic method for detecting SVCV. However, this method is time-consuming, reagent-intensive, and involves prolonged amplification steps, which increases the risk of contamination and may result in false-positive results [8]. Therefore, developing an improved diagnostic method that addresses these challenges is essential for enabling efficient and accurate disease diagnosis.

In this study, we demonstrated that one-step semi-nested RT-PCR showed a higher detection sensitivity than that of the two-step semi-nested RT-PCR method developed by Stone et al. [7]. This improved diagnostic approach was validated through comparative sensitivity and specificity analyses against currently listed WOAH methods for SVCV detection. Ultimately, we propose the adoption of this revised protocol in future WOAH diagnostic manuals.

## 2. Materials and Methods

### 2.1. Cell Culture, Virus Propagation, and Titration

Epithelioma papulosum cyprini (EPC; ATCC CRL-2872, Manassas, VA, USA) cells were cultured at 20 °C in minimum essential medium (MEM; Gibco, Billings, MT, USA), supplemented with 10% fetal bovine serum (FBS; Gibco) and 1% antibiotic-antimycotic (Gibco). The SVCV-A strain (subgroup Ia, GenBank accession no. MG663514.1) [9] was propagated in EPC cells at 20 °C. Virus-containing supernatants were collected via centrifugation at 6000× *g* for 10 min at 4 °C after cytopathic effects (CPE) appeared, aliquoted, and then stored at −80 °C until use. Virus titer was calculated using the Behrens–Kärber method [10].

### 2.2. Cell Culture Titration

EPC cells (1 × 10^6^ cells/mL) were seeded at 100 μL/well in 96-well plates with MEM containing 2% FBS and 1% antibiotic-antimycotic. The plates were incubated overnight at 20 °C to enable cell attachment. SVCV stock solution was serially diluted tenfold and inoculated at 25 μL/well. The plates were incubated at 20 °C and monitored for CPE for 2 weeks. All experiments were conducted in triplicate.

### 2.3. RNA Preparation for Sensitivity Comparisons

For the comparison of detection limits, RNA was extracted from the SVCV-A stock solution (Ia, 1 × 10^7.8^ TCID_50_/mL) using the QIAamp Viral RNA Mini Kit (Qiagen, Hilden, Germany), per the manufacturer’s instructions. Viral RNA was eluted in 20 μL AVE buffer and serially diluted tenfold (10^−1^–10^−6^) in diethylpyrocarbonate (DEPC)-treated water.

**Figure 1:**
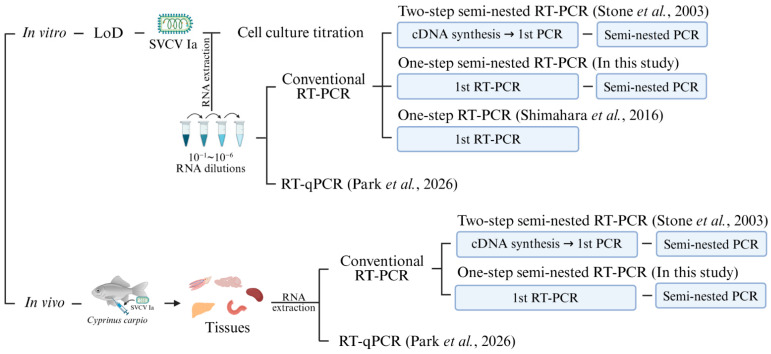
Schematic overview of the in vitro limit-of-detection (LoD) assay and in vivo detection of spring viremia of carp virus (SVCV) Ia in common carp (*Cyprinus carpio*) using reverse transcription PCR (RT-PCR)-based methods [7,11,12]. Created in BioRender: https://BioRender.com/ (accessed on 23 October 2025).

For in vivo sample detection, RNA was extracted from 30 mg common carp tissue using the RNeasy Mini Kit (Qiagen, Hilden, Germany) and eluted in 50 μL RNase-free water. RNA from the viral stock solution was used for one- and two-step semi-nested RT-PCR [7], one-step RT-PCR [11], and quantitative RT-PCR [12]. A schematic summarizing the experimental design is shown in Figure 1.

### 2.4. Reverse Transcription Quantitative Polymerase Chain Reaction

RT-quantitative PCR (RT-qPCR) was conducted using a dual-probe assay developed by Park et al. [12] (Table 1). The reaction mixture contained 15 μL SVC Master Mix, 4 μL SVC Oligo, 1 μL Internal Positive Control, and 5 μL RNA template. RT was conducted at 50 °C for 15 min. The PCR profile included initial denaturation at 95 °C for 5 min, followed by 45 cycles at 95 °C for 10 s and 55 °C for 20 s. Samples with Ct values < 42.91 were considered positive. Copy numbers were calculated using a standard curve generated by Park et al. [12].

### 2.5. Conventional Reverse Transcription PCR Methods

#### 2.5.1. Two-Step Reverse Transcription Polymerase Chain Reaction [7]

Two-step RT-PCR was conducted using the WOAH guidelines. Complementary DNA (cDNA) synthesis was conducted using the QuantiTect Reverse Transcription Kit (Qiagen, Hilden, Germany). The genomic DNA (gDNA) removal step was initially performed in a total reaction mixture volume of 14 µL (2 µL 7× gDNA Wipeout buffer, 11 µL DEPC-treated water, and 1 µL RNA), followed by the RT step. The reaction mixture for cDNA synthesis included Quantiscript Reverse Transcriptase, RT buffer, and SVCV R2 primers. PCR amplification was conducted using the AllTaq Master Mix Kit (Qiagen, Hilden, Germany) with SVCV F1 and R2 primers. The cycling conditions were 95 °C for 2 min; 35 cycles at 95 °C for 1 min, 55 °C for 1 min, 72 °C for 1 min, and final extension at 72 °C for 10 min.

#### 2.5.2. One-Step Reverse Transcription Polymerase Chain Reaction (This Study)

One-step RT-PCR, which combines cDNA synthesis and PCR into a single reaction, was conducted using the primer set (SVCV F1 and R2) of Stone et al. The QIAGEN^®^ OneStep RT-PCR Kit (Qiagen, Hilden, Germany) was used per the manufacturer’s instructions in the total volume of 25 µL reaction mixture containing RT-PCR buffer, deoxyribonucleotide triphosphate mix, enzyme mix, primers, RNA, and DEPC-water. Thermal cycling conditions included 50 °C for 30 min, 95 °C for 15 min, 35 cycles of 95 °C for 1 min, 55 °C for 1 min, 72 °C for 1 min, and final extension at 72 °C for 10 min.

#### 2.5.3. Nested Polymerase Chain Reaction

Semi-nested PCR was conducted using the SVCV F1 and R4 primers [7] following the same cycling conditions as those described for the first PCR step. Products were analyzed via 1.5% agarose gel electrophoresis. They were stained with ethidium bromide and visualized on Gel Doc UV or QIAxcel systems for in vitro and in vivo samples, respectively.

#### 2.5.4. One-Step Reverse Transcription Polymerase Chain Reaction

The one-step RT-PCR method developed by Shimahara et al. [11] was conducted using a QIAGEN^®^ OneStep RT-PCR Kit (Qiagen, Hilden, Germany). The reaction conditions were as follows: RT at 50 °C for 30 min; initial denaturation at 95 °C for 15 min; followed by 40 cycles at 94 °C for 15 s, 56 °C for 30 s, 68 °C for 1 min, and a final extension at 68 °C for 10 min. PCR products were electrophoresed and visualized as described in Section 2.5.3.

### 2.6. In Vivo Sample Detection

Juvenile common carp (average length 12 ± 3 cm, average weight 27 ± 2 g) were obtained from a farm in Asan, Chungcheongnam-do (Republic of Korea). The kidney, spleen, and liver tissues were screened for bacterial (*Aeromonas hydrophila* and *A. salmonicida*) and viral pathogens (Koi herpesvirus and SVCV) using PCR to ensure good health, confirming that all samples were negative.

Eighteen carp were intraperitoneally injected with 100 μL of a serially diluted SVCV stock solution to achieve non-lethal doses ranging from 10^3^–10^5^ TCID_50_/mL. A negative control group (conprising six carp) was injected with phosphate-buffered saline. The fish were maintained at 13 ± 1 °C and monitored for 2 weeks. The brain, gill, kidney, liver, and spleen tissues were collected (30 mg each) and used as unknown samples for one- and two-step semi-nested RT-PCR and RT-qPCR.

### 2.7. Comparison of Reproducibility Between Laboratories

For inter-laboratory reproducibility testing, 10 koi carp tissue samples (eight SVCV-positive and two SVCV-negative) were prepared for each laboratory. The samples were sent to the National Institute of Fisheries Science (WOAH Reference Laboratory for VHSV, Pusan, Republic of Korea). RNA was extracted using the RNeasy Mini Kit (Qiagen, Hilden, Germany), according to the manufacturer’s instructions. In both laboratories, the one-step semi-nested RT-PCR assay was performed in triplicate on all samples, including both negative and positive controls, by a single operator.

## 3. Results

### 3.1. Detection Limits Using Cell Culture and Molecular-Based Detection Methods

CPE was observed up to a 10^−5^ dilution in the virus-infected supernatants of EPC cell cultures with a detection rate of 66.67%. Similarly, RT-qPCR, using a dual-probe system, detected specific SVCV genes at the 10^−5^ dilution level. The one-step RT-PCR method of Shimahara et al. [11] also showed detection limits at 10^−5^ (Table 2, Supplementary Figure S1A). Conversely, the two-step semi-nested RT-PCR method developed by Stone et al. [7] had a detection limit of a 10^−2^ dilution (Table 2, Supplementary Figure S1B). Notably, one-step semi-nested RT-PCR using the primer set by Stone et al. [7] showed improved detection sensitivity at the 10^−5^ dilution, similar to that of the highest sensitivity methods tested (Table 2, Supplementary Figure S1C).

### 3.2. Comparison of Spring Viraemia of Carp Virus Detection Sensitivity Using Fish Tissue Samples

The two-step semi-nested RT-PCR method detected SVCV in 84.17% of the 120 reactions, whereas the one-step semi-nested RT-PCR method showed an increased detection rate of 91.67% (Table 3). All samples from the negative control group were consistently negative for both RT-PCR methods, indicating high specificity (Table 4).

### 3.3. Reproducibility

To evaluate inter-laboratory reproducibility, 10 carp tissue samples were analyzed via the one-step semi-nested RT-PCR assay in two independent laboratories. Identical positive and negative results were obtained in both laboratories for all samples (Table 5).

## 4. Discussion

The WOAH Manual for Aquatic Animal Diagnostics provides internationally accepted standards to ensure legal and scientific validity in aquatic animal trade and is aligned with the requirements of the World Trade Organization [3,13]. According to the WOAH guidelines, confirmation of SVC requires virus isolation in cell culture, gene-based detection methods, and nucleotide sequence analysis [3]. The present study provides a comprehensive comparison of the detection sensitivities of these diagnostic approaches.

The cell culture method demonstrated the highest sensitivity, detecting viral presence up to the 10^−5^ dilution, consistent with previous reports identifying cell culture as the gold standard for SVCV detection [4,12]. Molecular diagnostic methods, such as the RT-PCR assay developed by Shimahara and the dual-probe qRT-PCR developed in our previous study, showed comparable detection sensitivities, each detecting viral RNA to the 10^−5^ dilution [11,12]. In contrast, the two-step RT-PCR method by Stone et al. [7] exhibited approximately 1000-fold lower sensitivity, detecting viral RNA only up to the 10^−2^ dilution [7,8]. In comparison, the one-step semi-nested RT-PCR method devised in this study improved sensitivity by about 1000-fold compared with the conventional two-step method, emphasizing the need to strengthen current WOAH diagnostic guidelines [5].

The relatively low sensitivity of the traditional two-step semi-nested RT-PCR is likely due to reduced nucleic acid input following cDNA synthesis and primer degeneracy, both of which can reduce amplification specificity [8,11]. Nevertheless, Stone’s method was originally designed to amplify a genomic region encompassing SVCV genogroups Ia–Id, II, III, and IV, thereby enabling broad phylogenetic characterization of SVCV isolates. This broad coverage makes the method suitable for comprehensive SVCV detection compared with the one-step RT-PCR method by Shimahara et al. [7,11,14]. By adopting the Stone et al. [7] primer set within a one-step semi-nested RT-PCR framework, the present study enhances detection sensitivity while maintaining genotyping capability.

Rapid and accurate detection of SVCV in fish tissue is essential for border quarantine and disease management to prevent its transboundary spread [6]. In the present study, and consistent with the in vitro findings, the one-step semi-nested RT-PCR method showed an approximately 7.5% higher detection rate than the two-step method when applied to in vivo clinical samples, supporting its practical utility in field diagnostic applications. 

Ten carp tissue samples (eight SVCV-positive and two negative) were analyzed using the one-step semi-nested RT-PCR assay in two independent laboratories. As shown in Table 5, the positive/negative detection patterns were completely concordant between laboratories. This 100% agreement demonstrates the assay’s reproducibility and supports its applicability in both university laboratory and WOAH reference laboratory.

Additionally, the two-step semi-nested RT-PCR method requires multiple handling steps, increasing the risk of cross-contamination and false positives [8]. Notably, the two-step semi-nested RT-PCR protocol by Stone et al. [7] was developed more than two decades ago [7]. Therefore, transitioning to a streamlined, more sensitive one-step semi-nested RT-PCR protocol aligns well with contemporary diagnostic standards.

In this study, we redesigned the WOAH-recommended semi-nested RT-PCR framework into a one-step semi-nested RT-PCR format while preserving the broad genogroup coverage of the Stone et al. [7] primer set. The assay was systematically evaluated using cell culture titration quantitative RT-PCR, both in vitro and in vivo. Furthermore, complete inter-laboratory concordance between the university laboratory and the WOAH reference laboratory operating under the ISO/IEC 17025 quality system underscores not only the assay’s high analytical sensitivity, but also its practical robustness and reproducibility—aspects not comprehensively evaluated in previous SVCV molecular diagnostics studies.

In contrast to our recently published work [12], which focused primarily on applying a newly designed primer set for SVCV detection using real-time PCR, the present study optimizes the diagnostic workflow itself. In addition, real-time PCR does not provide genotyping information. 

Through integrating analytical sensitivity testing, in vivo diagnostic performance assessments, and inter-laboratory reproducibility, specifically using the Stone et al. [7] primer set, this study provides a robust and improved molecular diagnostic platform. Collectively, these features represent the core novelty of this work and support its potential adoption as an updated international standard for SVCV detection, contributing to enhanced disease management and improved compliance with international trade regulations.

## 5. Conclusions

The one-step semi-nested RT-PCR assay presented in this study provides a practical and improved diagnostic tool for SVCV, offering higher sensitivity and reduced contamination risk while preserving the broad genogroup coverage and genotyping capability of the original Stone et al. method. These advantages support its potential inclusion in international diagnostic guidelines, the WOAH Aquatic Manual, which would strengthen SVCV surveillance, improve disease control efforts, and help minimize economic losses in the aquaculture industry.

## Figures and Tables

**Table 1:** PCR primers and conditions used in this study.

			Primer Set	Oligonucleotide Sequence (5′ → 3′)	Product Size (bp)
Conventional PCR	Stone et al. [7]	1st	F1	TCTTGGAGCCAAATAGCTCARRTC	714
			R2	AGATGGTATGGACCCCAATACATHACNCAY	
		2nd	R4	CTGGGGTTTCCNCCTCAAAGYTGY	606
	Shimahara et al. [11]		SVCV-G1	TGAAGAYTGTGTCAATCAAGTC	369
			SVCV-G2	GCGARTGCAGAGAAAAAGTG	
RT-qPCR [12]	M gene		F	GAAGTCAAAAGGTACTCCTCCC	
			R	TTCAACATGGTACCTCATCCA	
			Probe	[FAM] TTACGAGGAGACACT [i-EBQ] GGCGACTGC [Phosphate]	
	N gene		F	GACCATTGAAGAGGTGTTCACATG	
			R	CGGCATGTAAGACGTGCTTTTATCT	
			Probe	[FAM] CTGCAGAGTG [i-EBQ] AAGTCGCGGATGAGTTAGT [Phosphate]	
	IPC		F	TGATAGTGGATACGTCTGTTTAGC	
			R	CTCGTCGGCTCTTTCCATC	
			Probe	[Cyanine5] ACCAGACACA [i-EBQ] CGCTCACACCTCCC [Phosphate]	

**Table 2:** Detection limits using cell culture and molecular-based detection methods.

Dilutions	Cell Culture	RT-qPCR	Shimahara et al. [11]	Stone et al. [7]	
				One-Step Semi-Nested RT-PCR	Two-Step Semi-Nested RT-PCR
10^−1^	6/6	6/6	6/6	6/6	6/6
	(100%)	(100%)	(100%)	(100%)	(100%)
10^−2^	6/6	6/6	6/6	6/6	4/6
	(100%)	(100%)	(100%)	(100%)	(66.67%)
10^−3^	6/6	6/6	6/6	6/6	0/6
	(100%)	(100%)	(100%)	(100%)	(0%)
10^−4^	6/6	6/6	6/6	4/6	0/6
	(100%)	(100%)	(100%)	(66.67%)	(0%)
10^−5^	4/6	3/6	2/6	2/6	0/6
	(66.67%)	(50%)	(33.33%)	(33.33%)	(0%)
10^−6^	0/6	0/6	0/6	0/6	0/6
	(0%)	(0%)	(0%)	(0%)	(0%)

Data are presented as positive replicates/total replicates with % values indicating the percentage of positive replicates.

**Table 3:** Detection rates of molecular diagnostic methods for SVCV-positive carp tissues.

Num.	RT-qPCR	One-Step Semi-Nested RT-PCR	Two-Step Semi-Nested RT-PCR
1	2/2	3/3	3/3
2	2/2	3/3	2/3
3	2/2	3/3	3/3
4	2/2	3/3	3/3
5	2/2	3/3	3/3
6	2/2	3/3	1/3
7	2/2	3/3	2/3
8	2/2	3/3	3/3
9	2/2	3/3	3/3
10	2/2	3/3	3/3
11	2/2	3/3	3/3
12	2/2	3/3	3/3
13	2/2	1/3	0/3
14	2/2	3/3	3/3
15	2/2	3/3	3/3
16	2/2	3/3	3/3
17	2/2	3/3	3/3
18	2/2	3/3	2/3
19	2/2	3/3	3/3
20	2/2	3/3	3/3
21	2/2	3/3	3/3
22	2/2	1/3	1/3
23	2/2	3/3	3/3
24	2/2	1/3	0/3
25	2/2	3/3	3/3
26	2/2	3/3	3/3
27	2/2	1/3	0/3
28	2/2	3/3	3/3
29	2/2	3/3	3/3
30	2/2	3/3	3/3
31	2/2	0/3	0/3
32	2/2	3/3	3/3
33	2/2	3/3	3/3
34	2/2	3/3	3/3
35	2/2	3/3	3/3
36	2/2	3/3	3/3
37	2/2	3/3	3/3
38	2/2	3/3	3/3
39	2/2	3/3	3/3
40	2/2	3/3	3/3
Total	80/80	110/120	101/120
	(100%)	(91.67%)	(84.17%)

Data are presented as positive replicates/total replicates with % values indicating the percentage of positive replicates.

**Table 4:** Detection results of molecular diagnostic methods for SVCV-negative carp tissues.

Num.	RT-qPCR	One-Step Semi-Nested RT-PCR	Two-Step Semi-Nested RT-PCR
1	0/2	0/3	0/3
2	0/2	0/3	0/3
3	0/2	0/3	0/3
4	0/2	0/3	0/3
5	0/2	0/3	0/3
6	0/2	0/3	0/3
7	0/2	0/3	0/3
8	0/2	0/3	0/3
9	0/2	0/3	0/3
10	0/2	0/3	0/3
Total	0/20	0/30	0/30
	(0%)	(0%)	(0%)

Data are presented as positive replicates/total replicates, with % values indicating the percentage of positive replicates.

**Table 5:** Inter-laboratory comparison of one-step semi-nested RT-PCR.

Num.	Laboratory 1	Laboratory 2
1	3/3	3/3
2	3/3	3/3
3	3/3	3/3
4	3/3	3/3
5	0/3	0/3
6	3/3	3/3
7	3/3	3/3
8	3/3	3/3
9	0/3	0/3
10	3/3	3/3
Total	24/30	24/30
	(80%)	(80%)

Laboratory 1, university laboratory at which the present study was conducted (Sunmoon University, Asan, Republic of Korea); Laboratory 2, National Institute of Fisheries Science (WOAH Reference Laboratory for VHSV, Pusan, Republic of Korea). Data are presented as positive replicates/total replicates, with % values indicating the percentage of positive replicates.

## Data Availability

The original contributions presented in this study are included in the article/Supplementary Materials. Further inquiries can be directed to the corresponding author.

## Supplementary Materials

The following supporting information can be downloaded at: https://www.mdpi.com/article/10.3390/microorganisms13122727/s1, Figure S1: Gel electrophoresis results of one-step semi-nested RT-PCR using RNA extracted from 10-fold serial dilutions of the SVCV-A (subgroup Ia) stock.

## Abbreviations

The following abbreviations are used in this manuscript:

cDNA	Complementary DNA
CPE	Cytopathic effects
DEPC	Diethylpyrocarbonate
ELISA	Enzyme-linked immunosorbent assay
EPC	Epithelioma papulosum cyprini
FBS	Fetal bovine serum
gDNA	Genomic DNA
IFAT	Indirect fluorescent antibody testing
MEM	Minimum essential medium
RT-PCR	Reverse transcription polymerase chain reaction
RT-qPCR	Reverse transcription quantitative polymerase chain reaction
SVC	Spring viremia of carp
SVCV	Spring viremia of carp virus
WOAH	World Organization for Animal Health
