# Supplementary material for: Improved Detection Sensitivity of Spring Viremia of Carp Virus by Substituting a Two-Step with a One-Step Nested Reverse Transcription Polymerase Chain Reaction Method
Source: Microorganisms. 2025 Nov 29;13(12):2727. doi: 10.3390/microorganisms13122727 (PMC12734977; doi:10.3390/microorganisms13122727)
Supplement: Supplementary file 1 [file microorganisms-13-02727-s001.zip › microorganisms-3974370-supplementary.pptx]

## Slide 1
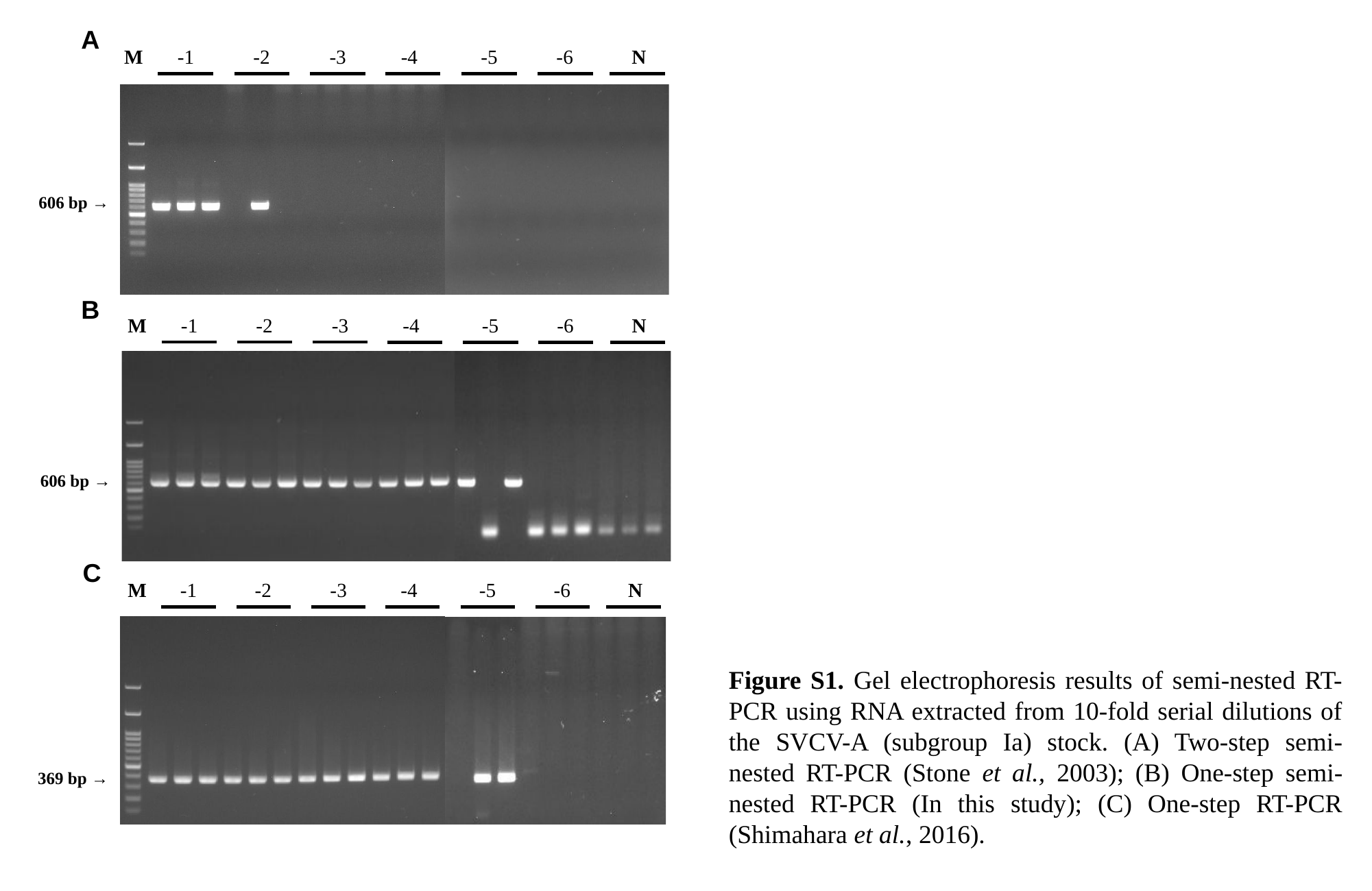

A
M
-1
-2
-3
-4
-5
-6
N
606 bp →
B
M
-1
-2
-3
-4
-5
-6
N
606 bp →
C
M
-1
-2
-3
-4
-5
-6
N
Figure S1. Gel electrophoresis results of semi-nested RT-PCR using RNA extracted from 10-fold serial dilutions of the SVCV-A (subgroup Ia) stock. (A) Two-step semi-nested RT-PCR (Stone et al., 2003); (B) One-step semi-nested RT-PCR (In this study); (C) One-step RT-PCR (Shimahara et al., 2016).
369 bp →
